# Positive attitude towards life and emotional expression as personality phenotypes for centenarians

**DOI:** 10.18632/aging.100456

**Published:** 2012-05-21

**Authors:** Kaori Kato, Richard Zweig, Nir Barzilai, Gil Atzmon

**Affiliations:** ^1^ Ferkauf Graduate School of Psychology, Yeshiva University, Bronx, NY 10461, USA; ^2^ Weill Cornell Medical College, Yeshiva University, Bronx, NY 10461, USA; ^3^ Institute for Aging Research and the Nathan Shock Center at Albert Einstein College of Medicine at Yeshiva University, Bronx, NY 10461, USA

**Keywords:** personality, measurement, Five-Factor Model, centenarians, aging

## Abstract

Centenarians have been reported to share particular personality traits including low neuroticism and high extraversion and conscientiousness. Since these traits have moderate to high heritability and are associated with various health outcomes, personality appears linked to bio-genetic mechanisms which may contribute to exceptional longevity. Therefore, the present study sought to detect genetically-based personality phenotypes in a genetically homogeneous sample of centenarians through developing and examining psychometric properties of a brief measure of the personality of centenarians, the Personality Outlook Profile Scale (POPS). The results generated two personality characteristics/domains, Positive Attitude Towards Life (PATL: optimism, easygoing, laughter, and introversion/outgoing) and Emotional Expression (EE: expressing emotions openly and not bottling up emotions). These domains demonstrated acceptable concurrent validity with two established personality measures, the NEO-Five Factor Inventory and Life Orientation Test-Revised. Additionally, centenarians in both groups had lower neuroticism and higher conscientiousness than the US adult population. Findings suggest that the POPS is a psychometrically sound measure of personality in centenarians and capture personality aspects of extraversion, neuroticism, and conscientiousness, as well as dispositional optimism which may contribute to successful aging.

## INTRODUCTION

Living to be 100 years of age is still a rare event. According to the US Census Bureau, there are approximately 53,000 centenarians in the US which remain only .2% of the population [1]. The number of centenarians has been rapidly increasing at a rate of 8% per year in the US [2]. Some suggest that centenarians may carry biological markers of successful aging since by and large they have delayed or escaped major illnesses [3]. However, less is known regarding factors which contribute to diversity in successful aging/longevity in centenarians [4].

Among many factors associated with longevity, personality has been linked to health outcomes and longevity [5]. Personality refers to a stable set of cognitive, motivational, social, and emotional traits and behavioral patterns, which is influenced by familial history, genetic predisposition, environment, and socio-cultural factors [6]. The NEO Personality Inventory-Revised (NEO-PI-R) and NEO-Five Factor Inventory (NEO-FFI) are personality measures developed for longitudinal studies of personality and aging [7] and are based on the Five Factor Model (FFM) which represents traits including neuroticism/emotional stability, extraversion, openness to experience, agreeableness, and conscientiousness. Select FFM traits have been associated with favorable health outcomes and longevity in older adults [5, 8-10].

Additionally, several studies have suggested that centenarians also share particular personality traits. The Georgia Centenarian Study reported that centenarians have a lower level of neuroticism and higher levels of extraversion, competence (a facet of conscientiousness), and trust (a facet of agreeableness) than the mixed-age US population [9]. Similarly, Japanese cognitively intact female centenarians were reported to have higher levels of extraversion and conscientiousness than the Japanese middle-aged and older adults [11]. Further, the Swedish Centenarian Study [12] showed that centenarians appeared more easygoing, relaxed, capable, and efficient than the Swedish mixed-age population.

The personality – longevity relationship has been further explored by examining pathways through which personality may influence health outcomes. Achieving 100 years of age seems to have a very strong genetic influence [13], and the genetic contribution has been found to be the largest in the oldest old population [14]. Personality may represent underlying genetic and neurophysiological mechanisms which may directly affect health outcomes [15, 16]. Additionally, certain personality traits shared by centenarians such as low neuroticism and high extraversion and conscientiousness [9, 11] may also have strong heritable/genetic components [16]. Distel and colleagues [17] reported that heritability estimates of FFM personality traits such as neuroticism, conscientiousness, and extraversion were 43%, 43%, and 47% respectively, suggesting that these traits are highly heritable. Similarly, the offspring of centenarians have been reported to show a lower level of neuroticism and a higher level of extraversion than the general population [18].

Second, personality traits (e.g., neuroticism, an enduring tendency to experience negative emotions) may affect health outcomes through mediating processes such as emotion regulation [19]. Specifically, depression, which may be viewed as a result of emotion dysregulation, has been reported to be a significant predictor for negative health outcomes and mortality [20-22], and neuroticism has been found to be an important risk factor for depression [19] and early mortality [23]. Further, some researchers argue that prolonged negative affect (e.g., depression and anxiety) is linked to disease susceptibility through physiological changes in SNS (sympathetic nervous system), HPA (hypothalamic-pituitary-adrenal) axis, stress hormones, blood pressure, metabolism, and immune function [19, 23-26]. Therefore, mediators linked to personality traits, such as chronic emotion dysregulation may have an adverse impact on health outcomes through their effect on physiological/biological function.

Third, personality may influence health outcomes through affecting an individual's choice of health-related behaviors [5, 12, 27]. Conscientiousness has been tied to longevity, and research suggests that people with high conscientiousness tend to practice healthy behaviors (e.g., engaging in physical exercise) and avoid risky health behaviors (e.g., excessive drinking and smoking) [28-31].

In summary, favorable personality characteristics have been associated with positive health outcomes in late life and longevity through various mechanisms. Less is known regarding whether genetically homogenous groups of centenarians share particular personality characteristics. Practical constraints to assessing the personality of centenarians (e.g., cognitive/sensory impairment) have also raised the need for a brief measure of personality. Therefore, this study developed a brief measure of personality characteristics in centenarians namely, the Personality Outlook Profile Scale (POPS), through examining the construct and internal consistency of the POPS (scale development study), as well as the concurrent validity of the POPS with the NEO-FFI, and LOT-R (validation study). To increase our ability to detect genetically-based personality phenotypes, the study targeted a genetically homogeneous sample of Ashkenazi Jewish centenarians.

## RESULTS

### Demographic Characteristics of the Participants

Demographic, cognitive, and personality characteristics of centenarians are presented in Table 1. The participants in the scale development study (N = 243) had a mean age of 97.6 ±2.79 and were predominantly female (75%). Years of education ranged from 0 to 25 years with a mean level of 12.7±3.89 years. The MMSE mean score was 19.8±11.95. The participants of the self-report group in the validation study (n = 19) had a mean age of 99.8 ±2.87 and were predominantly female (68%) with a mean score of 5.63±1.54 in the MIS-T, indicating that all were cognitively intact. The participants in the informant-report group of centenarians (n = 26) had a mean age of 100±2.27 and were predominantly female (69%). The mean scores and standard deviations (SD) of personality measures in the validation study, the population means of the NEO-FFI based on the normative data (derived from a representative sample of the US population) [7], and the mean score of the LOT-R based on a mixed age group (age 36 to 82 years with a mean age of 64.3 years) [32] are also presented in the Table 1. Centenarians in both self- and informant-report groups tended to be optimistic, easygoing, and outgoing and to consider laughter as an important part of their life, based on the raw mean scores of items on the POPS (ranging from 3.34 to 4.09 in the self-report group and from 3.68 to 4.21 in the informant-report group on a scale of 1 through 5). Further, the results of t-tests to compare mean scores of the NEO-FFI traits in the current sample to the normative population mean scores [7] found that participants in the self-report group showed lower mean scores of neuroticism, t(18) = −3.81, p < .01, as well as higher mean scores of extraversion, t(18) = 3.03, p < .01, agreeableness, t(18) = 3.82, p < .01, and conscientiousness, t(18) = 2.42, p < .05, than the US population means. Participants in the informant-group also showed lower mean scores of neuroticism, t(25) = −.2.24, p < .05, and higher mean scores of conscientiousness, t(25) = 2.88, p < .01, than the US population means. The results of t-tests to compare the mean score of the LOT-R in the current sample to the mean score of a mixed age group [32] also showed that participants in the informant-report group had a higher mean score of dispositional optimism, t(25) = 2.60, p < .05, than the mean score of the mixed age group.

**Table 1:** Demographic, Cognitive, and Personality Characteristics of Centenarians

Groups	Scale Development study	Validation study		US Population/Mixed Age Group
		Self-Reports	Informant-Reports	
Sample Size	N = 243	n = 19	n = 26	
Demographic Variable	Mean (SD)	Mean (SD)	Mean (SD)	Mean (SD)
Age	97.6 (2.79)	99.8 (2.87)	100 (2.27)	
Gender				
Males (%)	62 (25%)	6 (32%)	8 (31%)	
Females (%)	181 (75%)	13 (68%)	18 (69%)	
MMSE (score)	19.8 (11.95)	N/A	N/A	
MIS-T (score)		N/A	5.63 (1.54)	N/A
POPS PATL (z-score)	0.03 (0.70)	−.03 (0.42)	−.01 (0.60)	
Optimism	3.35 (1.26)	4.21 (0.86)	3.88 (0.82)	
Easygoing	3.34 (1.26)	3.68 (1.16)	3.19 (1.13)	
Laughter	4.09 (0.96)	4.17 (0.96)	4.29 (0.99)	
Introversion/Outgoing	3.71 (1.01)	3.68 (1.20)	3.65 (1.29)	
POPS EE (z-score)	0.02 (0.49)	0.00 (0.80)	0.00 (0.92)	
Bottle Up Emotions	2.62 (0.95)	2.53 (0.96)	2.77 (1.07)	
Express Feelings Openly	2.43 (0.88)	2.68 (1.06)	2.46 (1.07)	
NEO-FFI (score)				
Neuroticism	N/A	11.16 (9.06)	13.81 (11.97)	19.07 (7.68)
Extraversion	N/A	33.84 (8.85)	31.04 (8.59)	27.69 (5.85)
Openness to Ex	N/A	26.53 (8.93)	26.88 (8.15)	27.03 (5.84)
Agreeableness	N/A	40.00 (8.17)	34.54 (9.50)	32.84 (4.97)
Conscientiousness	N/A	39.21 (8.34)	39.81 (9.28)	34.57 (5.88)
LOT-R (score)				
Dispositional Optimism	N/A	17.11 (5.08)	17.50 (4.59)	15.16 (4.05)

*Note*. MMSE = Mini Mental Status Exam; MIS-T = Memory Impairment Screen - Telephone; PATL = Positive Attitude Towards Life; EE = Emotional Expression; NEO-FFI = NEO Five Factor Inventory; LOT-R = The Life Orientation Test-Revised; POPS = Personality Outlook Profile Scale.

### Scale Development Study: The Generation of the POPS Personality Domains

The results of a principal component analysis generated three personality domains based on nine items which were highly inter-correlated (Tables 2): Domain 1: Positive Attitude Towards Life (PATL: Easygoing, Optimism, Laughter, and Introversion/Outgoing); Domain 2: Emotional Expression (EE: Bottle Up Emotions and Express Feelings Openly); Domain 3: Transcendent Outlook (TO: Spirituality and Relaxation). Findings based on an examination of internal consistency (Cronbach's alpha) showed alpha levels of .65 (PATL), .63 (EE), and .50 (TO). Two domains, PATL and EE, were selected for the POPS measure due to their adequate internal consistency.

**Table 2:** Principal Component Analysis of Personality Characteristics with Varimax Rotation

Variable	Component 1	Component 2	Component 3
Spiritual	0.22	−0.04	**−0.78**
Relaxation	0.07	0.01	**−0.84**
Bottle Up Emotions	0.12	**0.83**	−0.08
Express Feelings	−0.03	**0.82**	0.03
Creative	0.45	−0.06	0.09
Easygoing	**0.64**	0.28	0.20
Optimistic	**0.64**	0.18	0.20
Laughter	**0.71**	−0.06	0.01
Introverted/Outgoing	**0.62**	−0.51	0.00

### Validation Study: The Concurrent Validity of the POPS with Personality Measures

The validation study examined the concurrent validity of the POPS with the NEO-FFI and LOT-R. The results of the bivariate correlations (Tables 3) demonstrated that, in the self-report group of centenarians, PATL was positively associated with extraversion (r = .82, p < .01) and conscientiousness (r = .53, p < .05). EE was also negatively correlated with neuroticism (r = −.51, p < .05). In the informant report group of centenarians, PATL was negatively correlated with neuroticism (r = −.52, p < .01), and positively correlated with extraversion (r = .74, p < .01) and dispositional optimism (r = .69, p < .01). Additionally, EE was positively associated with extraversion (r = .45, p < .05). There were no other significant associations between the POPS domains and other personality traits/measures in both groups.

**Table 3:** Correlations between Personality Domains, the LOT-R, NEO-FFI

Groups		Self-Reports (n=19)		Informant-Reports (n=26)	
Variable		PATL	EE	PATL	EE
NEO-FFI:	Neuroticism	−0.35	−.51*	−.52*	−0.21
	Extraversion	.82**	0.26	.74**	.45*
	Openness	0.36	−0.23	0.12	−0.19
	Agreeableness	0.05	0.07	0.17	0.04
	Conscientiousness	.53*	0.11	0.17	−0.01
LOT-R:	Optimism	0.25	0.25	.69**	0.35

*Note*. PATL = Positive Attitude Towards Life; EE = Emotional Expression; NEO-FFI = NEO Five Factor Inventory; LOT-R = The Life Orientation Test-Revised; POPS = Personality Outlook Profile Scale.

*p < .05 **p < .01 ***p < .001

## DISCUSSION

The purpose of the present study was to detect genetically-based personality phenotypes through developing a brief measure of personality of centenarians, the Personality Outlook Profile Scale (POPS) and examining its construct, internal consistency (scale development study), and concurrent validity with the NEO-FFI and LOT-R (validation study) in a sample of Ashkenazi Jewish centenarians. The scale development study generated two domains: domain 1, Positive Attitude Towards Life (PATL: optimism, easygoing, laughter, and introversion/out-going); domain 2, Emotional Expression (EE: expressing emotions openly and not bottling up emotions). These domains evidenced adequate levels of internal consistency. Findings of the validation study demonstrated moderate to high associations between the POPS and select personality traits from the NEO-FFI and LOT-R. Specifically, PATL was highly associated with extraversion in both groups. PATL was also moderately associated with conscientiousness in the self-report group, and moderately to highly associated with dispositional optimism and neuroticism (negative correlation) in the informant-report group. EE was moderately and negatively associated with neuroticism in the self-report group and was moderately associated with extraversion in the informant-report group. There-fore, findings suggest that the POPS has acceptable psychometric characteristics and measures personality aspects of extraversion, neuroticism, and possibly conscientiousness, as well as dispositional optimism.

Particularly noteworthy findings were the favorable personality characteristics of centenarians in this study compared to the US population means, and personality characteristics of centenarians found in this study seem to be consistent with findings from previous studies. Specifically, centenarians in both self- and informant-report groups in the validation study showed lower mean scores of neuroticism and higher mean scores of conscientiousness than the US population means. These results coincided with several studies that have identified that centenarians may share higher levels of extraversion and conscientiousness, and low levels of neuroticism [9, 11]. However, whether centenarians have maintained these particular personality traits across their lifespan remains unknown since this question has not been addressed longitudinally, although some age-associated changes in personality in older adults have been reported [7, 33].

The present study has a number of limitations. Although the use of a genetically and environmentally homogeneous sample was an effective method to identify genetically-based personality phenotypes, the use of a homogeneous group potentially reduces the generalizability of this study's findings. However, because Ashkenazi Jewish individuals are not expected to have more longevity than other groups and their survival/causes of death are similar to those reported in the general Caucasian US population [34], we do not expect that their personality phenotypes are unique, and thus our findings may well be relevant to other populations. Another limitation of this study is that the sample size of the validation study was small because most participants in the original study were unable to participate due to their health issues or mortality. There were some methodological challenges in assessing centenarians’ personality characteristics using the self-report measures include their sensory impairment, cognitive constraints, and physical disabilities. In the scale development study, the Centenarian Questionnaire was completed by participants with the assistance of a family member in order to increase the reliability of participants’ responses to the questionnaire. Also, in the validation study, the informant-report group was used in addition to the self-report group. Although sufficient levels of the agreement between self- and informant-reports on personality measures have been demonstrated [35, 36], informant-reports may not represent the true personality characteristics of the participants.

In summary, the findings of the present study demonstrated that the POPS displays adequate psychometric characteristics as a brief measure of personality in centenarians and measures aspects of neuroticism, extraversion, and possibly conscientiousness, as well as optimism. This study adds to a growing body of knowledge which suggests that centenarians may share particular personality characteristics and suggests that genetically-based aspects of personality may play an important role in achieving positive health outcomes and exceptional longevity. Future research should continue to examine further reliability and validity (e.g., discriminant validity) of the POPS as the development of brief and robust measures of personality, validated for the oldest old, in order to stimulate further research regarding successful aging in this rapidly growing population.

## METHODS

### Participants

As part of the Longevity Genes Project, Ashkenazi Jewish centenarians (age 95 to 107), who were living independently at 95 years of age as a reflection of good health, were recruited [34]. The use of the Ashkenazi Jews in this study was due to their genetic and environmental homogeneity which is an effective method to facilitate genetic association. Survival and causes of death in the Ashkenazi Jewish population are similar to those reported in the general Caucasian US population, and participants also had relatively similar socio-cultural, economical, and educational back-grounds. The study's sampling, recruitment, and procedures have been described previously [34, 37-39]. Within the archival data consisting of N = 396 Ashkenazi Jewish centenarians (age 95 to 107), 153 individuals were excluded due to missing questionnaire responses. These individuals were not different from the participants who were included in the analysis. Thus, N=243 participants were included in the scale development study. For the validation study, cognitive screening measures were employed to reduce variance due to cognitive impairment. N=210 cognitively intact centenarians, who scored 24 points or higher on the Mini-Mental State Exam (MMSE) were selected. Of those, 102 subjects were still alive at the time of this study, and 26 centenarians and their children were available to participate. Most were unable to participate in the study due to their health issues and practical constraints. Of these, 7 centenarians in the self-report group were excluded due to health issues and cognitive impairment (scores of lower than four points on the Memory Impairment Screen by Telephone) [40]. Therefore, participants in the validation study included centenarians (n=19) in the self-report group and their children (n=26) in the informant-report group.

### Procedures

In the Longevity Genes Project, the Centenarian Questionnaire was filled out by participants with the assistance of their children in the event that participants had difficulty responding to questions in a reliable manner (e.g., due to mild cognitive and sensory/motor impairment). A single research nurse administered the MMSE to each participant. In the validation study, MIS-T [40], POPS, NEO-FFI [7], and LOT-R [32] were administered to centenarians through an in-person or phone interview (self-reports). Their children were also asked to complete the personality measures through an in-person or phone interview or by mail (informant-reports).

### Measures

#### The Centenarian Questionnaire and Personality Outlook Profile Scale (POPS)

The 98-item Centenarian Questionnaire, developed by the Longevity Genes Project, measures participants’ personal demographics, personality, health/medical history, and health-related behaviors [41]. This questionnaire had served as the basis for the development of the POPS. Initially, 11 items of the Centenarian Questionnaire, which reflected centenarians’ lifetime personality and related behaviors, were selected as candidate items for the POPS. These items were developed using a rational content-based approach and derived from extant longevity research and theoretical concepts. Based on a principal component analysis, six of the original eleven candidate items were selected, and two robust components were identified: Positive Attitude Towards Life (PATL: optimism, easygoing, laughter, and introversion/outgoing) and Emotional Expression (EE: expressing emotions openly and not bottling up emotions). A composite score of each domain is based on a mean z-score of respective personality items for each domain. Higher scores reflect more favorable personality traits.

#### NEO-Five Factor Inventory (NEO-FFI)

The NEO-Five Factor Inventory (NEO-FFI) is a self- and informant-report 60-item inventory which is a short-version of the NEO-PI and measures five broad dimensions of personality traits. Each personality trait scale is comprised of 12 likert-type items rated on a five-point scale. Total scale scores are derived by summing responses of the 12 items for each trait. Higher scores indicate higher levels on a given trait. The NEO-FFI has demonstrated correlations with the NEO-PI ranging from .75 to .91 [7].

#### The Life Orientation Test-Revised

The Life Orientation Test-Revised (LOT-R) is a measure of dispositional optimism, which examines generalized expectancies about future life events. Likert-type items are rated on a 0 (disagree strongly) to 4 point scale (agree strongly). Total scores are derived by the summation of scores on six items; 4 items are filler items and not scored. Scores range from 0 to 24, with 24 reflecting extreme optimism, with a mean score of 15 in a mixed-age adult sample. The LOT-R has adequate levels of internal consistency, test-retest reliability, convergent/discriminant validity, and validity for older adults [32].

#### Mini Mental Status Exam (MMSE)

The Mini Mental Status Exam (MMSE) is a standard 30-item measure validated as a screening tool for cognitive impairment. The MMSE has demonstrated acceptable levels of internal consistency, test-retest reliability, and concurrent validity. A standard cutoff score of ≥24 on the MMSE26 for intact cognitive function was required for inclusion in the validation study [42, 43].

#### The Memory Impairment Screen by Telephone (MIS-T)

The MIS-T is a telephonically administered adaptation of the Memory Impairment Screen (MIS), a four-item screening test for dementia which assesses an individual's verbal memory based on the delayed free and cued recall trials. The MIS-T is a valid screening tool for Alzheimer's type dementia in older adults. Total scores are derived by the summation of the correct responses. A cut-off score of four has adequate levels of sensitivity (.80) and specificity (.96) to detect Alzheimer's dementia [40, 44], and a score of four or above was required for inclusion in the validation study.

### Statistical Methods

Analyses were completed with the statistical software, SPSS Statistics (Version 17.0). In the scale development study, a principal component analysis was conducted to generate the domains of the POPS. Cronbach's alpha was computed to test the internal consistency of each domain. In the validation study, the concurrent validity of the POPS with other measures was examined using Pearson's correlation coefficients.
